# Usefulness of Intravital Multiphoton Microscopy in Visualizing Study of Mouse Cochlea and Volume Changes in the Scala Media

**DOI:** 10.3389/fneur.2017.00332

**Published:** 2017-07-31

**Authors:** Hyun Mi Ju, Sun Hee Lee, Tae Hoon Kong, Seung-Hae Kwon, Jin Sil Choi, Young Joon Seo

**Affiliations:** ^1^Laboratory of Smile Snail, Yonsei University Wonju College of Medicine, Wonju, South Korea; ^2^Department of Otorhinolaryngology, Yonsei University Wonju College of Medicine, Wonju, South Korea; ^3^Department of Bio-imaging, Korea Basic Science Institute, Chuncheon, South Korea

**Keywords:** cochlea, anatomy, Meniere’s disease, multiphoton microscopy, scala media

## Abstract

Conventional microscopy has limitations in viewing the cochlear microstructures due to three-dimensional spiral structure and the overlying bone. But these issues can be overcome by imaging the cochlea *in vitro* with intravital multiphoton microscopy (MPM). By using near-infrared lasers for multiphoton excitation, intravital MPM can detect endogenous fluorescence and second harmonic generation of tissues. In this study, we used intravital MPM to visualize various cochlear microstructures without any staining and non-invasively analyze the volume changes of the scala media (SM) without removing the overlying cochlear bone. The intravital MPM images revealed various tissue types, ranging from thin membranes to dense bone, as well as the spiral ganglion beneath the cochlear bone. The two-dimensional, cross-sectional, and serial z-stack intravital MPM images also revealed the spatial dilation of the SM in the temporal bone of pendrin-deficient mice. These findings suggest that intravital MPM might serve as a new method for obtaining microanatomical information regarding the cochlea, similar to standard histopathological analyses in the animal study for the cochlea. Given the capability of intravital MPM for detecting an increase in the volume of the SM in pendrin-deficient mice, it might be a promising new tool for assessing the pathophysiology of hearing loss in the future.

## Introduction

Despite its small size, the cochlea has a complex three-dimensional helical structure, consisting of the modiolus, a central pillar of spongy bone, and the spiral lamina winding around it. These structures include spaced filled with fluid [scala vestibuli, scala media (SM), and scala tympani] and the organ of Corti. The traditional microscopic methods used for examining these cochlear microstructures involve the creation of paraffin-embedded tissue sections. However, the three-dimensional complex microstructure of the cochlea requires precise tissue manipulation for histological processing and viewing technically challenging (1). And many embedding sections may result in the loss of tissue antigenicity. In addition, those make it difficult to conduct immunochemical studies on sections. Frozen sections can retain the antigenicity of most tissues and can provide thicker sections that allow some depth of field (2). However, these issues can be overcome by imaging the cochlea *in vitro* with intravital multiphoton microscopy (MPM), which allows the visualization of cochlear microstructures by determining the exited endogenous fluorescence and second harmonic generation (SHG) of the explant cochlea.

In 1990, Denk et al. (3) at Cornell University pioneered two-photon microscopy, later referred to as intravital MPM. It proved to be a useful tool for detailed visualization of the microstructures of various unprocessed tissues, even at the subcellular levels, by using only intrinsic two-photon-excited fluorescence (TPEF) and SHG. The intrinsic endogenous fluorescence and SHG from tissues provide subcellular-resolution images with sufficient morphological details that render them diagnostically useful, as has been demonstrated with unprocessed biopsy specimens obtained from the human urinary bladder (4), ovary (5), gastrointestinal tract (6), lungs (7), and prostate (8). Moreover, the intravital MPM images provide information about tissue architecture, similar to that obtained using established histopathological diagnostic methods.

Ablation of the overlying bone is required to access the cochlear microstructures, especially the SM, which is located deep within the cochlear bone. Although conventional fluorescence microscopy provides excellent spatial resolution and allows selective quantification of cochlear changes, it requires the ablation of the overlying bone to access the cochlear structures and preparatory thinning of the cochlear lateral wall. Intravital MPM is a light microscopy technique that allows *in vivo* imaging up to a depth of 1 mm from the surface of some tissue specimens (9). Moreover, it overcomes the constraints posed by the scattering and absorption of photons in light microscopy by using as its excitation source near-infrared light at frequencies that have superior scattering characteristics. The application of two simultaneous photons with high frequency and low energy allows for more focused imaging confined to small volumes (10). Thus, intravital MPM eliminates the need for invasive tissue sampling and complex preparation, while enabling the volumetric visualization of a specimen. Various methods, such as laser-Doppler flowmetry (11, 12), micro-computed tomography (CT) (13, 14), and magnetic resonance imaging (15), have been applied to visualize the microstructures over the cochlear bone. However, these techniques reveal only the blood vessels over the cochlear bone or require the invasive use of contrast media to identify the microstructure of the SM. Intravital MPM can be used to visualize the SM over the cochlear bone without any contrast injection.

In this study, we first describe a new morphometric method for various cochlear microstructures by using intravital MPM without any contrast medium, and compare it with light microscopy and confocal microscopy. We then applied intravital MPM to visualize the volume changes of the SM without removing the overlying cochlear bone in the pendrin-deficient mouse, a pathologic model for endolymphatic hydrops. To our knowledge, this is the first report on the use of this approach for cochlear imaging.

## Materials and Methods

### Mice

Six male C57BL/6 mice for ototoxicity testing were involved and allowed free access to water and a regular mouse diet. They were kept at room temperature under a standard 12-h light/dark cycle for 1 week of acclimatization before the experiments. For comparing the changes of the SM, two normal (Pds+/+) and two pendrin-deficient mice (i.e., Pds-knockout; Pds−/−) were obtained from colonies bred from heterozygous mice kindly provided by Choi et al. (16). The animals were 5 weeks old and weighed approximately 18–25 g. The mice were anesthetized by intraperitoneal injection of 30 mg/kg tiletamine–zolazepam (Zoletil, 500 mg/vial; Virbac, Carros, France) and 10 mg/kg xylazine (Rompun; Rompun, Bayer Korea, Ansan, Korea). Animals underwent cardiac perfusion with phosphate-buffered saline (PBS: Dulbecco’s Formula Modified, ICN Biochemicals, England) before tissue harvest. After temporal bones were dissected, the bony shells of the cochlea and vestibule were removed in Cl-free physiologic saline. All experimental procedures performed in this study followed ethical guidelines for animal studies and were approved by the Institutional Animal Care and Use Committee of Yonsei University College of Medicine (IACUC No. YWC-150728-1). All experiments were performed in accordance with relevant guidelines and regulations.

### Ototoxic Drug Administration

The first injection for each mouse was given at the beginning of the daily light cycle. The three mice in the ototoxicity group received a subcutaneous injection of kanamycin (550 mg/kg; Sigma-Aldrich, Oakville, ON, Canada) dissolved in PBS, followed by an intraperitoneal injection of furosemide (130 mg/kg; Sigma-Aldrich, Oakville, ON, Canada) *via* the tail vein 30 min later (17). The three mice in the control group received a subcutaneous injection of saline, and followed by another saline injection 30 min later intraperitoneally. Mice showing signs of severe dehydration or other serious illness were killed. All animals were monitored by trained animal care technologists supervised by a veterinarian. All animals survived the drug administration.

### Hearing Testing

The thresholds of Pre-test auditory brainstem response (ABR) were measured 24 h prior to the first drug injection. Each animal was gently anesthetized with an intraperitoneal injection of ketamine (100 mg/kg; Yuhan Corporation, Seoul, Korea) and xylazine (1 mg/kg; Rompun; Korea Bayer, Ansan, Korea) and kept warm by using a heating pad. Under effective anesthesia, subdermal needle electrodes were positioned on the scalp vertex, ipsilateral posterior bulla, and contralateral posterior bulla for recording ABR. The clicks were used as a test stimuli using BioSigRP (Tucker-Davis Technologies). The stimulus intensity was decreased gradually in 5-dB steps, and the lowest sound level that caused this waveform was defined as the ‘threshold’. Post-test ABR thresholds were measured 14 days after kanamycin and furosemide administration. We confirmed that all three mice in the ototoxicity group showed higher thresholds over 50 dB than did the control mice (Figure S1 in Supplementary Material).

### Histological Assessment

The temporal bones were removed, and the apex of the cochlea, the round window, and the oval window were punctured. A fixative was perfused through the cochlear apex with 4% paraformaldehyde (Biosesang Seongnam, Korea), and then the sample was immersed in the fixative for 24 h at 4°C. Cochleas were decalcified by immersion in Calci-Clear Rapid (National Diagnostics, Atlanta, GA, USA) for 24 h, dehydrated in 30% sucrose (Sigma-Aldrich, Gillingham, UK) for 24 h, embedded in optimal cutting temperature compound (Leica, Bensheim, Germany), and sectioned at 5- to 2-μm thickness in a cryostat (Leica CM1850 Cryostat; Leica, Wetzlar, German). A standard hematoxylin and eosin (H&E) staining protocol was followed, with a 1- to 3-min incubation in hematoxylin and 30- to 60-s staining with eosin, before mounting the samples.

### *Ex Vivo* Intravital MPM Imaging on the Cryosections of Mouse Cochlea

**Figure 1:**
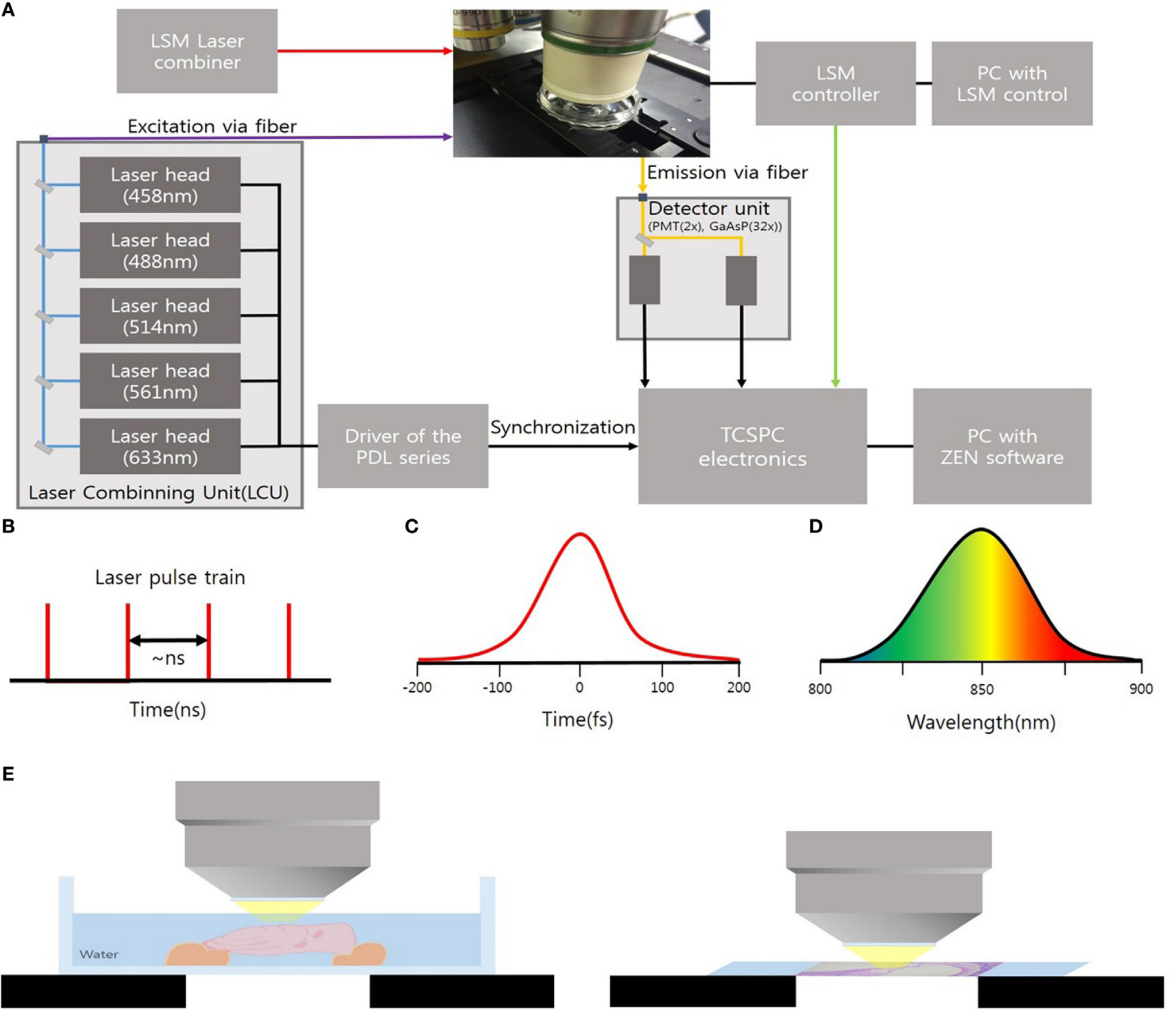
**(A)** Experimental setup to microscopy based on an upright microscope and LSM-780 NLO confocal scan head; AOM, acousto-optic modulator; T1, telescope; λ/4, quarter waveplate; G1/G2, galvanometer mirrors. **(B)** Ti:Sa Laser provides pulsed mode 80 MHz. **(C)** Pulses out of the laser typically have a <70 fs **(D)** of 8.7 nm. **(E)** Schematic showing *ex vivo* temporal bone imaging (left) and *ex vivo* imaging of the cryosections of the mouse cochlea (right) by using water-immersion lens.

The cryosections of mouse cochlea were visualized under the spectral imaging mode of a multiphoton laser microscope (LSM-780 NLO; Carl Zeiss, Jena, Germany) equipped with a MaiTai (690–1,040 nm) laser (Figure 1). Figure 1A shows an experimental apparatus. The system showed an upright microscope equipped with a confocal scan head (LSM-780 NLO, Carl Zeiss Microscopy GmbH, Germany). The excitation beam was provided by a mode-locked Ti: Sapphire laser (Spectra-Physics. Irvine, CA, USA) emitting <70 fs width pulses at the wavelength of 690–1,040 nm with the 80 MHz repetition rate. This laser system worked with a DeepSee for group velocity dispersion compensation and an acousto-optic modulator (AOM) for laser power attenuation. And the beam was coupled to the scan head after a collimating telescope (T1). Circular polarized light was used to avoid anisotropies for different fibril’s directions. To do this, we placed a broadband quarter wave plate (λ/4-Newport) in the laser beam in front of the microscope scanning head and moved the plate until the optical power after the objective lens no longer changes due to the rotation of the polarizer. The beam was focused onto the sample by a 40×/1.20 NA water immersion. The sample was exposed to an excitation wavelength ranging from 800 to 900 nm, and the output power of the laser was about 10 mW. Images were acquired using the ZEN software (Carl Zeiss). Spectral images were acquired using a 32-channel GaAsP detector, which collected photon signals in the selected wavelength range from 415 to 686 nm at an interval of 8.7 nm. The signals were then converted to a pseudo-colored image in which the color of each pixel matched the corresponding photoluminescence spectrum. The laser operating power was kept below 10 mW to minimize phototoxicity.

### *Ex Vivo* Intravital MPM Imaging of the Temporal Bone

The extracted temporal bones were placed in 2-ml microcentrifuge tubes containing 250–500 μl 4% paraformaldehyde diluted with 10 mM PBS at pH 7.4 and incubated at room temperature for 24 h. The sample was then placed between the bottom of the confocal glass bottom culture dish from which the cover and cover slip had been removed. Bone wax was filled in the gap between the cover and the bottom of the culture dish and the dish was filled with water (18). The temporal bone was placed on a microscopy examination table filled with water; the water-immersion lens was dipped into this water-containing dish for imaging. The apical and second turns of the cochlea were imaged using the spectral imaging mode of the LSM-780 multiphoton microscope at an excitation wavelength range of 800–900 nm. *In situ* spectral deconvolution imaging, in which ZEN software (Carl Zeiss) was used to acquire an image in spectral imaging mode and immediately deconvolved it into a signal corresponding to the reference spectrum. Background autofluorescence was discarded during the image acquisition period.

## Results

### Intravital MPM Imaging of Microstructures in the Sections of the Normal Cochlea

**Figure 2:**
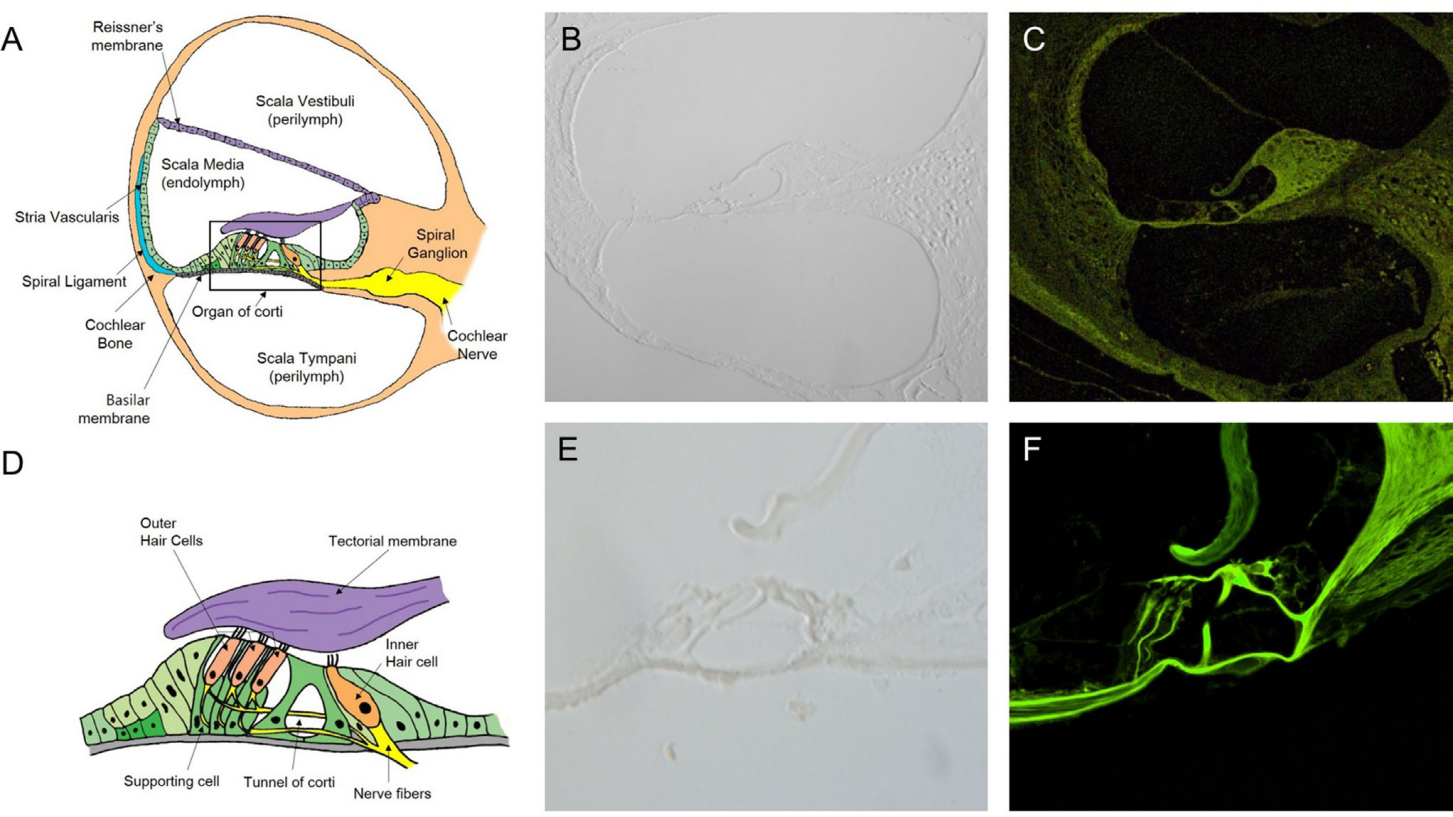
Intravital multiphoton microscopy (MPM) imaging of the microstructures in the sections of the normal cochlea. **(A)** A schematic cross section of the cochlea showing the three compartments (the scala vestibuli, scala tympani, and scala media), which are separated by two elastic partitions: Reissner’s membrane and the basilar membrane (BM). **(B)** A micrograph of a 2-mm-thick cryosection slide under light microscopy (200×) does not show the cochlear microstructures. **(C)** The intravital MPM image without any staining clearly shows the different cochlear microstructures. **(D)** A schematic of the organ of Corti, which is located above the BM, showing the inner and outer hair cells, as well as hairs attached to the flap called the tectorial membrane, which sits over the organ of Corti. **(E)** A micrograph of a 2-mm thick cryosection slide under light microscopy (400×). **(F)** At a higher magnification (400×), the intravital MPM image shows the organ of Corti in detail with green-endogenous autofluorescence and second harmonic generation.

In the normal cochlea, the space of the SM contains the organ of Corti, surrounded by the spiral ligament, stria vascularis, Reissner’s membrane (RM), and basilar membrane, and attached to the cochlear nerve tract and modiolus (Figure 2A). The organ of Corti consist of the tectorial membrane (TM), outer hair cells (OHC), inner hair cells (IHC), and supporting cells (Deiter cells, Hensen cells, and Claudius cells) (Figure 2D). We could not identify these microstructures in the views for the cryosection slides of the cochlea without H&E staining (Figures 2B,E). Moreover, in the confocal microscopy images, the unstained sections showed very weak autofluorescence. No cochlear microstructures could be visualized by confocal microscopy. Nevertheless, intravital MPM imaging allowed for the easy identification of cochlear microstructures without any staining. Autofluorescence generated over the range of 500 to 650 nm in two different channels as red and green fluorescence was recorded. Although autofluorescence provides enough low magnification (200×, Figure 2C) to discriminate the microstructures, a higher magnification (400×) could be more beneficial, as shown in Figure 2F.

**Figure 3:**
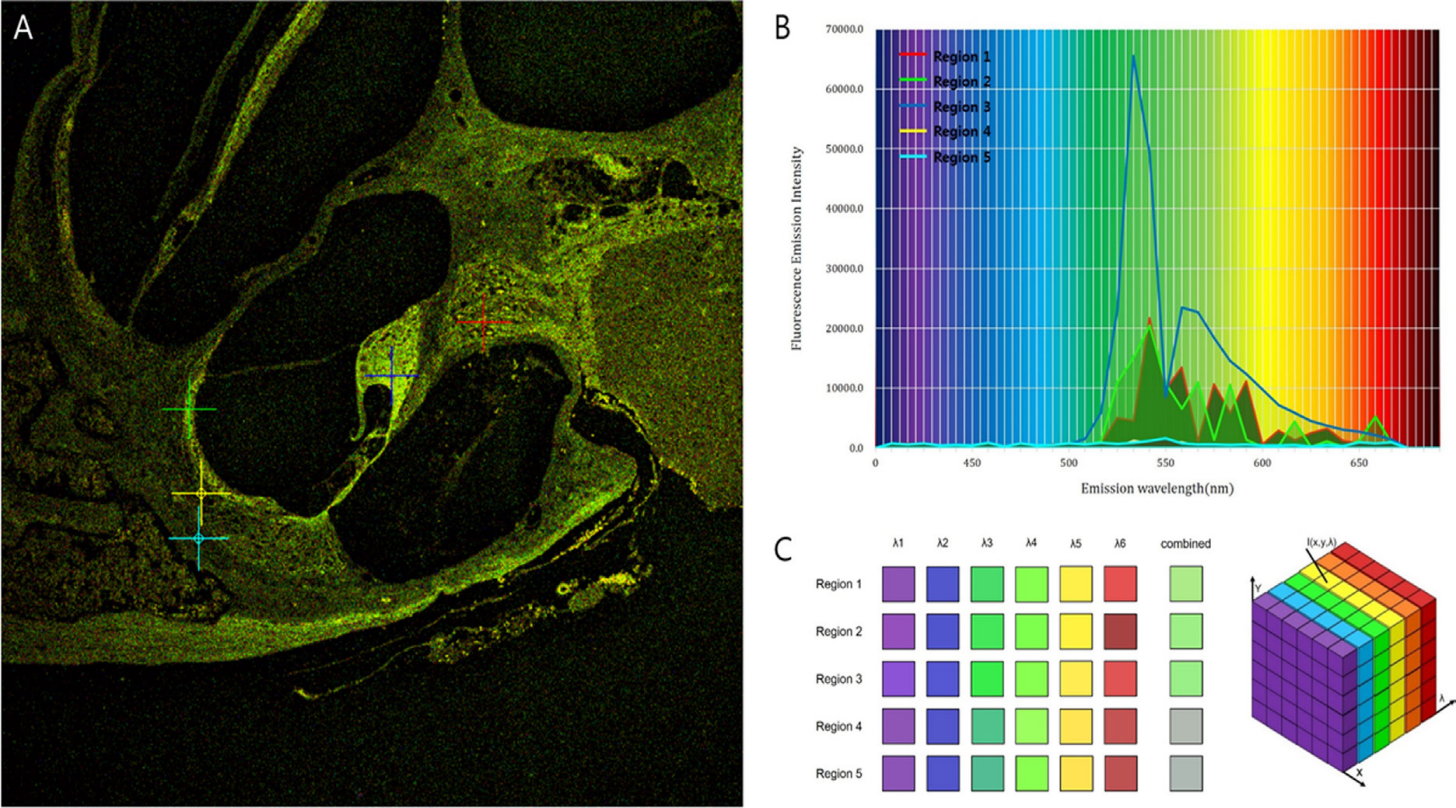
Different fluorescence emission spectra of each tissue in the cochlea. **(A)** The peaks of intensity are different for five microstructures: Region 1 (red), spiral ganglion; region 2 (green), stria vascularis; region 3 (blue), tectorial membrane; region 4 (yellow), spiral ligament; and region 5 (sky-blue), cochlear bone. **(B)** Excitation spectra of the five different tissues show that irrespective of their own peaks, most wavelengths exist in the range of 530–610 nm. **(C)** Although the five regions have different spectra, the combined spectral image stacks of the five microstructures explain why most of the tissues in the cochlea show similar distribution of green autofluorescence.

**Figure 4:**
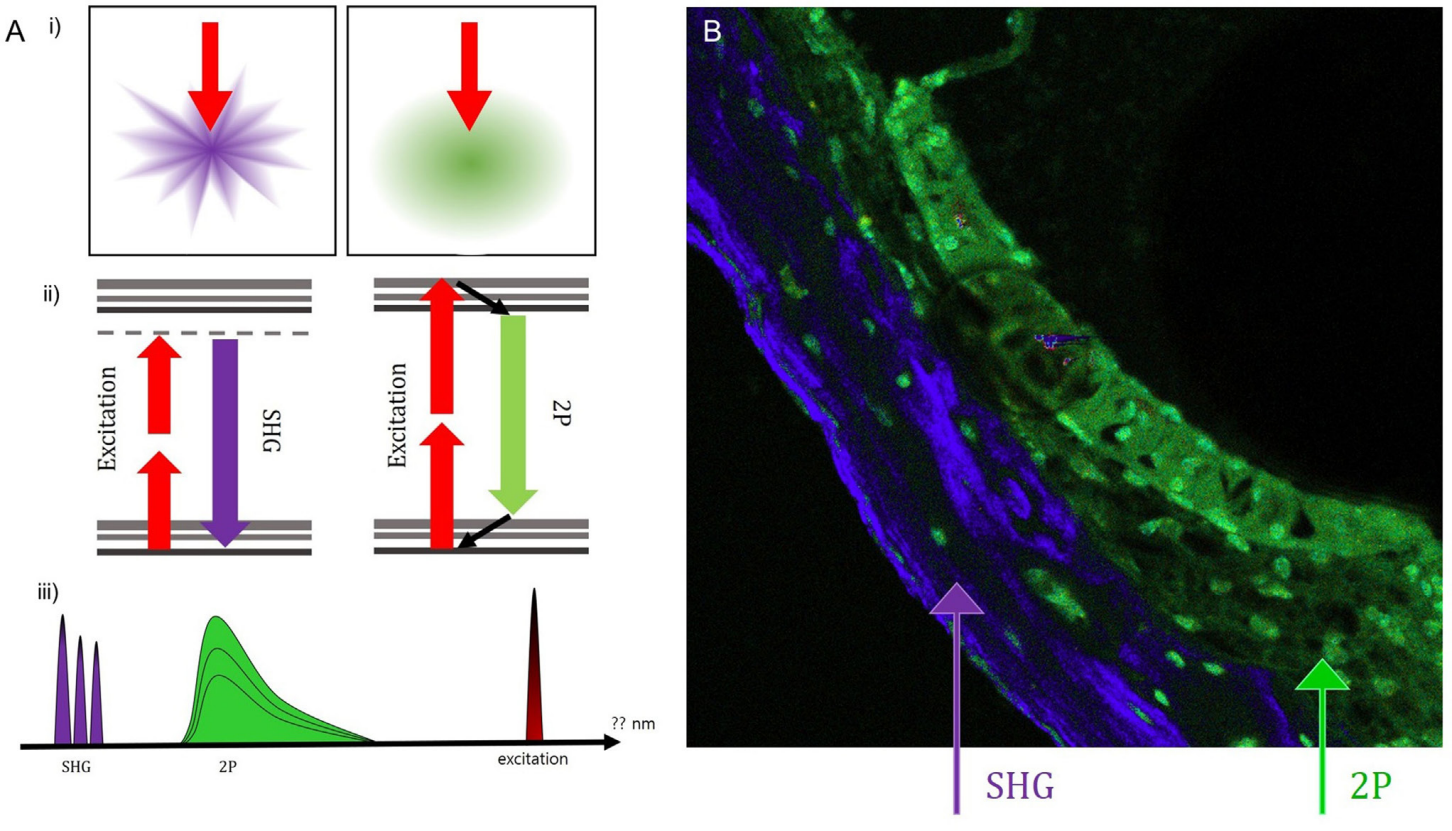
Using the pseudo-coloring option in the ZEN software (Carl Zeiss) according to the characteristics of different intensities, we could compare the green-endogenous autofluorescence and second harmonic generation (SHG) with intravital multiphoton microscopy. **(A)** (i) SHG is directional depending on the distribution and orientation of the non-linear dipoles, whereas general fluorescence is isotropically emitted. (ii) A diagram illustrating the different reactions in the absorption of two photons to excite the fluorescent molecule and the visible fluorescence emitted during relaxation. (iii) Detection of spectral ranges for excitation at 415–686 nm: half the excitation wavelength for SHG and two spectral bands for two-photon-excited fluorescence (TPEF). **(B)** Combined SHG/TPEF images of the cochlea clearly show the borders and even the nuclei of cells in the stria ligaments with pseudo-coloring; however, it is not easy to discriminate the borders between the stria vascularis, spiral ligament, and cochlear bone. SHG (violet), cochlear bone; TPEF (green), stria ligaments.

Intravital MPM did not show significantly different spectra of intrinsic fluorescence even in the excitation range from 800 to 900 nm from an argon ion laser with a power level of up to 10 mW and measurements were taken at the back focal plane of the objective. However, the peaks of intensity were different for five microstructures (Figure 3; region 1: spiral ganglion (SG); region 2: stria vascularis; region 3: TM; region 4: spiral ligament; and region 5: cochlear bone). The TM (blue line) had two high-intensity peaks of 65,353 at 546 nm and 23,496 at 573 nm (Table S1 in Supplementary Material). Although the other cochlear regions had their own peaks, most wavelengths were in the range of 530–610 nm. Discriminating the borders between the stria vascularis, spiral ligament, and cochlear bone was difficult. Using the pseudo-coloring option in the ZEN software (Carl Zeiss) according to the characteristics of different intensities, we could clearly discriminate the borders and even visualize the nuclei of cells in the stria ligaments (Figure 4).

### Intravital MPM Imaging of Microstructures in the Sections of the Cochlea with Ototoxicity

**Figure 5:**
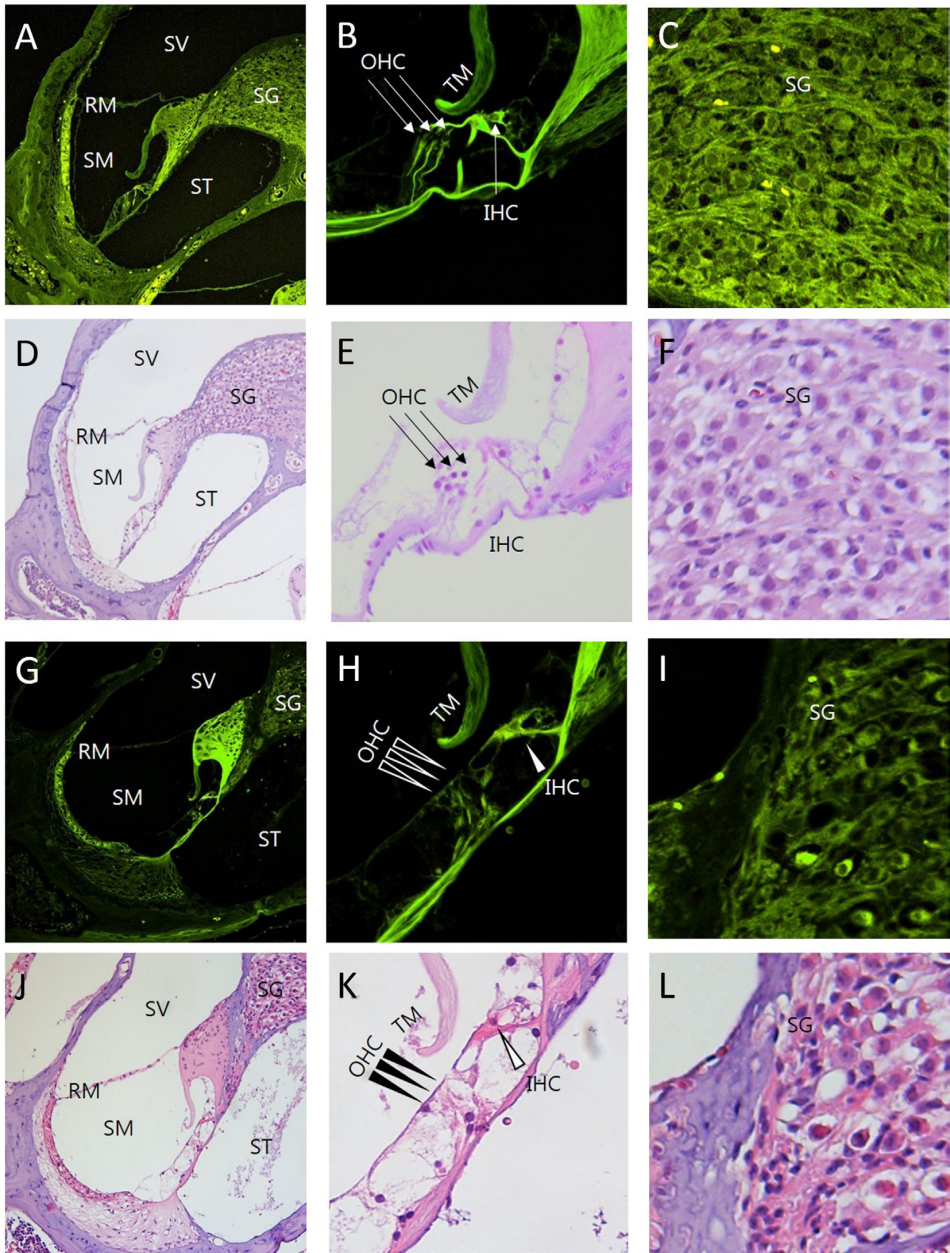
Comparison between intravital multiphoton microscopy (MPM) and conventional light microscopy in the sections of the cochlea exposed to ototoxicity. We compared the intravital MPM images without any staining in the cryosections from normal mice **(A–C)** and mice exposed to ototoxicity **(G–I)** with hematoxylin and eosin (H&E) staining of normal mice **(D–F)** and mice exposed to ototoxicity **(J–L)**. The images acquired after H&E staining and the intravital MPM images reveal similar morphological changes caused by ototoxicity in the cochlea, organ of Corti, and spinal ganglion. The organ of Corti **(B,E,H,K)** shows a clear cytoarchitecture. Note the appearance of the organ of Corti in a normal mouse **(B,E)** and the injuries to the IHC in the basal turn **(H,K)**: the arrows indicate the area without supporting cells, pillar elements, or hair cells [**(H,K)** IHC, open arrowheads; OHC, arrowheads]. Note the appearance of the SG in a normal mouse **(C,F)**. In contrast, the nerve fibers show diminished staining and decreased neuronal packing density in the SG **(I,L)** of a mouse exposed to ototoxicity. Intravital MPM can be successfully used to image the cochlea, and the cochlear structures show good correlation between the intravital MPM images without any staining and conventional light microscopic images with H&E staining. SV, scala vestibuli; SM, scala media; ST, scala tympani; RM, Reissner’s membrane; TM, tectorial membrane; BM, basilar membrane; SG, spiral ganglion; OHC, outer hair cells; IHC, inner hair cells.

The images of the H&E-stained sections and corresponding intravital MPM images revealed similar ototoxicity-induced morphological changes (Figure 5). The loss of OHC in the organ of Corti was higher in the cochlea exposed to the ototoxicity than the normal cochlea (Figures 5B,H). Extensive destruction of the OHC in the three-layer arrays, normal appearance of the IHC, and decreased count and vacuolization of the SG cells were typical initial characteristics of the cochlea exposed to ototoxicity (Figures 5C,I). Unlike light microscopy and confocal microscopy, intravital MPM imaging without staining allowed for the identification of changes in the various microstructures of the cochlea exposed to ototoxicity.

### *Ex Vivo* Intravital MPM Imaging of the SM Space in the Bone Overlying the Cochlea of the Normal and Pendrin-Deficient Mice

**Figure 6:**
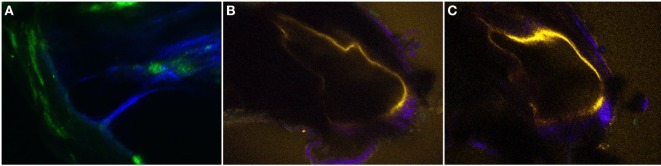
The two-dimensional cross-sectional **(A–C)** intravital multiphoton microscopy images (32-channel GaAsP) showing the internal structures of the extracted bone covering the intact temporal bone in the normal and pendrin-deficient mice. **(A)** In the normal cochlea, the scala media (SM) appears as a right-angled triangle. **(B,C)** The cochlea of a pendrin-deficient mouse shows a relatively large endolymphatic fluid-filled space, or dilated SM, in the different sections. The images in **(A–C)** could be reconstructed differently with pseudo-coloring according to different wavelengths.

**Figure 7:**
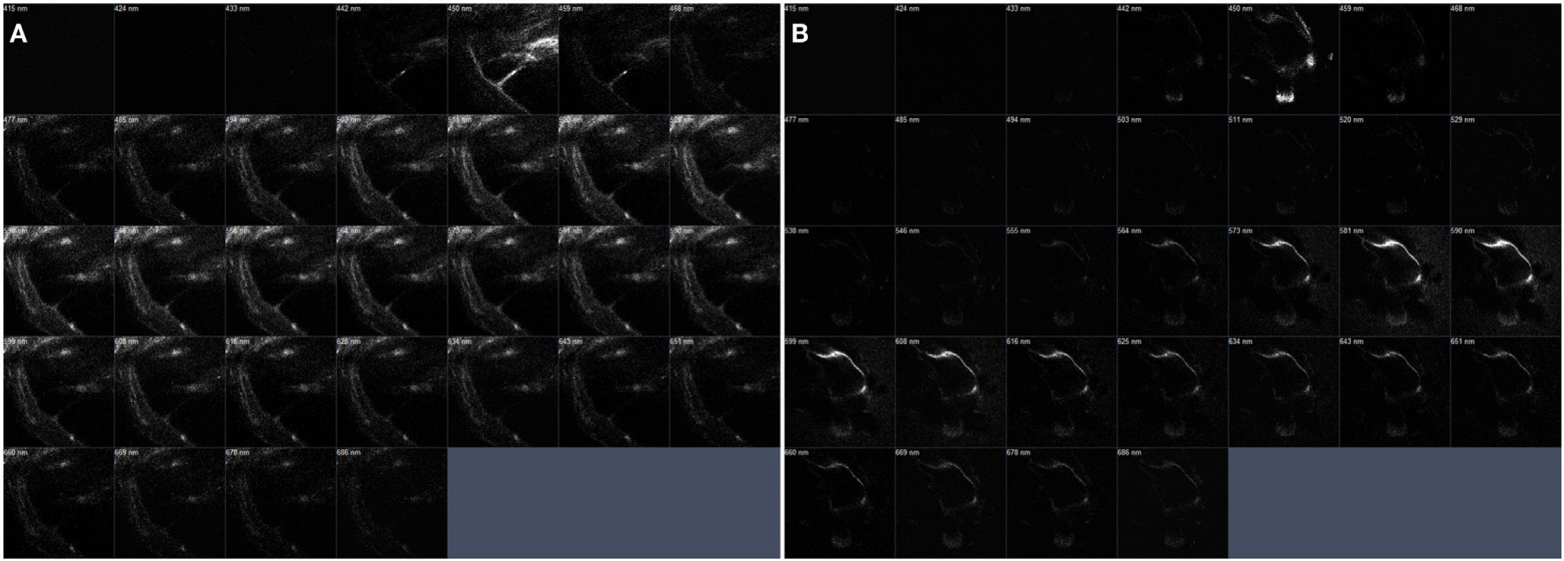
The serial z-stack intravital multiphoton microscopy images (32-channel GaAsp) of the internal structures of extracted bone covering intact cochlea. **(A)** The normal cochlea has the scala media (SM) shown as a right-angled. On the contrary. **(B)** The cochlea of pendrin knockout mouse confirms relatively large endolymphatic fluid-filled space, SM, at each different section.

Figures 6 and 7 show the two-dimensional cross-sectional and serial z-stack intravital MPM images of the internal structures of the extracted bone covering the intact cochlea. We compared the volumetric changes of the SM in normal and pendrin-deficient mice. Pendrin-knockout mice are models of endolymphatic hydrops, which shows spatial dilation of the SM. In the normal cochlea, the SM appears as a right-angled triangle (Figure 6A; Video S1 in Supplementary Material). However, in the cochlea of pendrin-knockout mouse, relatively large endolymphatic fluid-filled spaces are seen in different sections of the SM (Figures 6B,C; Video S2 in Supplementary Material). Reissner’s membrane with a swollen sharp turn bends into the scala vestibuli. The morphological features of RM in mice with endolymphatic hydrops were clearly shown in the intravital MPM images.

The bone of apical turn in cochlea was an optical area in Figure 6, but we could confirm the space in both apical and second turns of cochlea. The dilation of the SM could be clearly seen even at a relative shallow depth in the apical turn. The penetration depth of this wavelength in turbid media (such as biological tissues) varies from approximately 0.5 to 7 mm. Several linear structures in the apical turns exhibit high signal magnification (400×), which indicates the basilar and RMs. The stria vascularis and spiral ligament were well visualized as a distinct crescent-shaped structure adjacent to the promontory bone. The boundary between the bony labyrinths and the membrane labyrinths was clearly identified and a spirall ligament was observed. Clear identification of this structure made it possible to distinguish the overlying stria vascularis.

We were able to visualize various tissue types from Reissner’s membrane to high density bone and SG. However, the organ of Corti, which contains remarkably delicate hair cells, could not be discriminated from the surrounding tissues.

The original images (Figures 6A–C) could be reconstructed differently by using pseudo-coloring at various wavelengths (Figure 7). While the normal cochlea showed strong intensities at wavelengths ranging from 450 to 500 nm, the cochlea of pendrin-deficient mice showed strong intensities at wavelengths ranging from 580 to 620 nm. These differences could determine the different pseudo-coloring between the cochlea of normal and pendrin-deficient mice.

## Discussion

We describe a new method for microscopic analysis of the mammalian cochlea that offers several advantages over alternative histological preparations and allows for non-invasive spatial visualization of the SM without removing the cochlear bone. First, we performed *ex vivo* intravital MPM imaging on cryosections of unprocessed cochlea from normal mice and those exposed to ototoxicity. A comparison of the images acquired after H&E staining and intravital MPM images from the same region before. Following tissue processing, it was shown that two-photon excitation intrinsic fluorescence and SHG provide similar microstructural information, as in standard histopathology. Although the conventional histopathology could be cheap and an easy access tool, it could not evaluate the whole 3D spiral structure of cochlea. And the reissner’s membrane would be ruptured during manipulating the specimen. Definitely, there might be several advantages in cochlear study with using the intravital MPM. Our results demonstrate that, similar to H&E stating, two-photon-excited intrinsic fluorescence and SHG can provide enough structural and spectral contrast to identify the occurrence of ototoxicity in the cochlea. Intravital MPM could detect typical ototoxicity-related pathological changes in mouse microstructures, without the need for tissue processing. Furthermore, intravital MPM could be used to observe the space of the SM and to compare the pathologic changes occurring in this space, without having to remove the bone covering the unprocessed cochlea in pendrin-deficient mice.

Fluorescence is the emission of visible light after the substance has absorbed light or other electromagnetic radiation. Normally, the absorbed light gets a shorter wavelength and higher energy than the emitted light. This is known as single photon fluorescence applied in traditional fluorescence microscopy. In conventional confocal microscopy, upon excitation, the fluorophore molecule absorbs energy from a single photon and then releases energy as emitted photons. In contrast, intravital MPM excite the fluorophores in the specimen with pulsed longer wavelength light (19). In two-photon excitation, two photons must fill the phosphor simultaneously (within 10–18 s) in order to excite the phosphor to emit fluorescent photons of shorter wavelength and higher energy. This phenomenon is rare and can occur only in high photon density regions. This phenomenon makes intravital MPM more advantageous than conventional confocal microscopy (3, 20, 21). Intravital MPM is based on non-linear optical effects such as SHG. Owing to the intensity of the laser passing through a highly polarized material, SHG is generated at precisely half the wavelength of the original light. By using near-infrared lasers for two-photon excitation, the SHG can be kept in the range of the visible wavelength. Because many intrinsic biological structures, including collagen fiber, muscle, brain, and bone, induce this kind of effect, these structures can be visualized without labeling them with exogenous probes. Using intravital MPM, Pena et al. (22) provided significant insights into lung fibrosis; in their study, intravital MPM allowed for the separation of the inflammatory and fibrotic steps in the pathophysiology of lung fibrosis. In brain research, we studied nerve structure and function using intravital MPM imaging. Dendrites and spine can be imaged to study changes in shape and size. Changes in the axonal morphology of deep brain tissue could be determined by visualizing the activity of the neuronal population (23, 24). Our findings in the mouse cochlea show that the excited endogenous autofluorescence and SHG in intravital MPM images can provide useful information similar to histopathological analyses, without the need for tissue processing. We also demonstrate the utility of replacing histopathological analysis of the cochlea with intravital MPM without staining. Leake et al. (25) studied the effect of aminoglycoside ototoxicity, the first row of OHC first degenerated, then the second row, then the third row; the last cells to degenerate in a given region were the IHC. Moreover, the SG displayed different patterns of degeneration, early cell loss occurred in the middle of the cochlea. In our study, Intravital MPM images revealed these ototoxicity-induced changes in the cochlear organ of the mouse cochlea, the stria vascularis and the organ of the SG.

As long wavelength and low energy excitation light reduces photodamage and increases penetration depth, thereby enabling imaging of live specimens, intravital MPM has revolutionized development in biological imaging, it is thought (26). The most commonly used fluorophores in intravital MPM have excitation spectra in the range of 400–500 nm, whereas the laser used to produce TPEF emits in the wavelength range of 700–1,000 nm. In our study, we collected photon signals in the selected wavelength range from 415 to 686 nm after excitation using wavelengths ranging from 800 to 900 nm. By using these wavelengths, it was possible to discriminate the microstructures in the cochlear cryosections by pseudo-coloring. However, discriminating the stria ligaments between the stria vascularis and the cochlear bone can be difficult because these layers are closely attached to each other. Different spectra of wavelengths in different tissues (vessels, ligaments, bone, etc.) could be easily shown as different colors by using the ZEN software. We presume the spectra would differ according to differences in the density and laser absorption ability of cells. Intrinsic emissions from living tissues can yield different details that might ultimately prove useful in clinical diagnosis.

Another advantage of intravital MPM is the ability to obtain bright, high-resolution images from deep areas of tissues and organs. In this study, we have referred to these images as intravital MPM images for discriminating them from the cryosection images acquired using intravital MPM.

The intravital MPM can spatially limit the excitation region to the focal point of the objective lens and the objective lens concentrates photons in a very small region (9). This enables the visualization of structures without fixation or thin sectioning. The near-infrared laser used in the intravital MPM can absorb more or less penetrate than the visible light or UV light used in confocal microscopy and can deeply penetrate, so it has a depth of 100–1,000 μm in the body MPM. Depths less than 100 μm can be visualized using confocal microscopy (21). Visualizing deep cochlear bone tissue has inherent limitations because both visible light and infrared light are easily scattered by crystallized calcium phosphate in the bone matrix. However, the cochlear bone of the mouse is only 32–100 μm thick, which is thin enough to fall within the penetration range of the MPM in the body. Intravital MPM overcomes a number of problems associated with substantial histological artifacts due to fixation, decalcification, dehydration, and cochlear implantation. An entire cochlea can be imaged using intravital MPM within a couple of minutes, whereas the fixation, decalcification, paraffin embedding, sectioning, and imaging in histological analysis would typically take a week or more to complete. In addition, excitation with near-infrared lasers can minimize photobleaching, i.e., the destruction of fluorophores and phototoxicity-induced tissue damage. Optical coherence tomography could identify the histological confirmation of the cochlea (27). OCT is an imaging technique that utilizes the principle of interferometry to obtain images of tomographic images such as ultrasound and CT. Though the OCT is capable of depth-resolved imaging and its axial resolution is good, the scattering contrast of OCT lacks the molecular and biochemical specificity, while intravital MPM could detect the other fluorescent proteins in the live animal study. We thought that intravital MPM and OCT have complementary characteristics which make them good candidates for viewing the microstructures of cochlea in the future.

Pds−/− mice develop early-onset, profound deafness, and hence serve as a valuable model for studying auditory dysfunctions associated with pendrin deficiency. The lack of pendrin in Pds−/− mice leads to excessive dilation of the SM inside the cochlea. This is secondary to a change in the osmotic pressure environment of the endolymph caused by the absence of the anion transport function of pendlin (28). Our intravital MPM findings suggest that the volume of the SM was higher in Pds−/− mice than in Pds+/+ mice. Cochlear images of pendlin-deficient mice acquired without removing or thinning the cochlear bone showed expected composition and structural changes. Serial z-stack imaging could be used to calculate the overall volume of the SM. In addition, it would facilitate the analysis of structural changes in various locations. We could not evaluate the space of the SM in living mice by imaging only the cochlea because of artifacts due to heartbeats and difficulty in positioning the mice.

In future research it is necessary to evaluate whether intravital MPM can be used to detect cartilage soft tissue change *in vivo*. We believe that the visualization of the SM by intravital MPM will serve as a diagnostic tool for endolymphatic hydrops. However, since the ear cranial bone is thicker in humans, visualization through a circular window membrane will be necessary. The successful application of intravital MPM imaging in the temporal bone of mice suggests that it could become a promising new tool for assessing the pathophysiology of hearing loss.

## Author Contributions

YS carried out design of the study and performed the statistical analysis. HJ and TK participated in performing the animal experimental. SL, JC and S-HK performed the imaging with IVMPM. All authors read and approved the final manuscript.

## Conflict of Interest Statement

The authors declare that the research was conducted in the absence of any commercial or financial relationships that could be construed as a potential conflict of interest.

## Supplementary Material

Video S1 The serial z-stack views in the scala media of the normal mice cochlea.

Video S2 The serial z-stack in the scala media of the pendrin-knockout mice cochlea.
