# Supplementary figures and images for: Usefulness of Intravital Multiphoton Microscopy in Visualizing Study of Mouse Cochlea and Volume Changes in the Scala Media
Source: Front Neurol. 2017 Jul 31;8:332. doi: 10.3389/fneur.2017.00332 (PMC5535263; doi:10.3389/fneur.2017.00332)

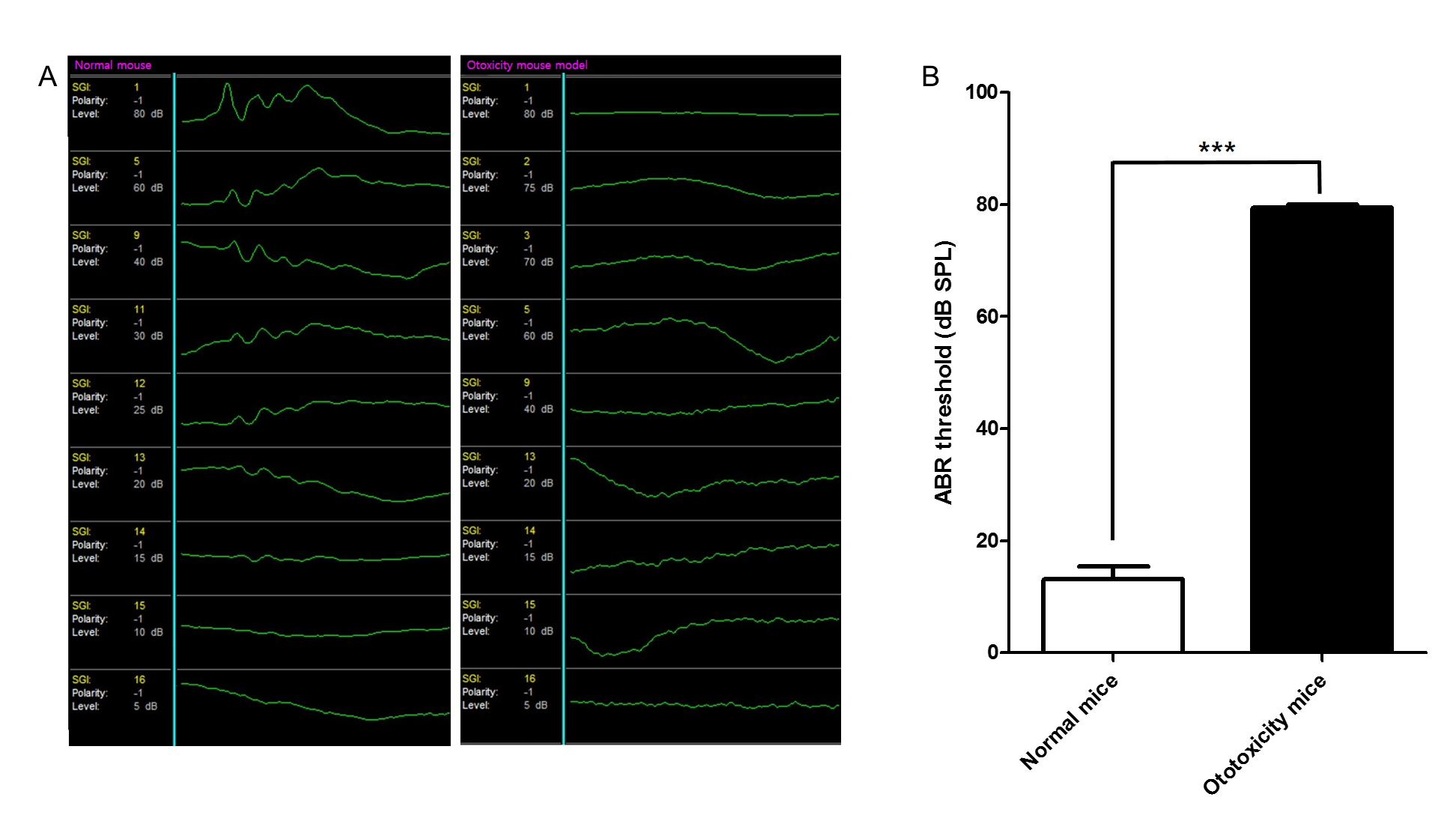

Supplement: Supplementary file 3 [file Image_1.JPEG]
