# Supplementary material for: Usefulness of Intravital Multiphoton Microscopy in Visualizing Study of Mouse Cochlea and Volume Changes in the Scala Media
Source: Front Neurol. 2017 Jul 31;8:332. doi: 10.3389/fneur.2017.00332 (PMC5535263; doi:10.3389/fneur.2017.00332)

 suppl. table 1. Different fluorescence emission spectra of each tissue in the cochlea.

| Emission wavelength  (nm) | **Region 1** | **Region 2** | **Region 3** | **Region 4** | **Region 5** |
| --- | --- | --- | --- | --- | --- |
| 415 | 717.0 | 22.0 | 142.2 | 742.6 | 752.8 |
| 424 | 697.0 | 215.0 | 127.6 | 611.8 | 623.6 |
| 433 | 654.0 | 362.0 | 229.4 | 764.6 | 783.3 |
| 442 | 439.0 | 74.0 | 152.9 | 412.4 | 450.0 |
| 450 | 382.0 | 381.0 | 556.7 | 460.0 | 518.0 |
| 459 | 344.0 | 93.0 | 298.1 | 385.7 | 432.0 |
| 468 | 714.0 | 311.0 | 298.5 | 771.7 | 810.4 |
| 477 | 289.0 | 23.0 | 237.1 | 279.7 | 286.1 |
| 485 | 809.0 | 385.0 | 338.5 | 751.0 | 741.1 |
| 494 | 337.0 | 143.0 | 341.8 | 485.3 | 467.1 |
| 503 | 616.0 | 337.0 | 360.9 | 505.7 | 481.3 |
| 511 | 540.0 | 427.0 | 574.9 | 675.1 | 751.6 |
| 520 | 468.0 | 366.0 | 1517.6 | 504.3 | 630.6 |
| 529 | 841.0 | 1075.0 | 5944.9 | 795.2 | 879.7 |
| 538 | 5085.0 | 10977.0 | 23111.6 | 273.8 | 709.0 |
| 546 | 4524.0 | 15014.0 | 65535.0 | 1441.4 | 893.1 |
| 555 | 21684.0 | 20468.0 | 49155.0 | 1020.0 | 1201.4 |
| 564 | 9852.0 | 10834.0 | 8470.6 | 979.3 | 1638.8 |
| 573 | 13438.0 | 6589.0 | 23496.1 | 1126.8 | 785.5 |
| 581 | 1159.0 | 10971.0 | 22716.4 | 498.8 | 632.0 |
| 590 | 10706.0 | 1376.0 | 18336.1 | 790.0 | 608.7 |
| 599 | 5849.0 | 10521.0 | 14514.7 | 520.9 | 471.4 |
| 608 | 11189.0 | 1383.0 | 12343.5 | 604.7 | 613.4 |
| 616 | 633.0 | 176.0 | 9770.2 | 658.4 | 409.1 |
| 525 | 2960.0 | 359.0 | 7175.3 | 570.6 | 427.7 |
| 634 | 1359.0 | 4444.0 | 5903.5 | 430.0 | 473.2 |
| 643 | 2511.0 | 212.0 | 4469.8 | 455.7 | 245.5 |
| 651 | 3307.0 | 1129.0 | 3761.7 | 575.9 | 505.3 |
| 660 | 1216.0 | 152.0 | 3095.3 | 313.0 | 361.7 |
| 669 | 809.0 | 1187.0 | 2728.7 | 809.3 | 938.2 |
| 678 | 530.0 | 5277.0 | 2103.5 | 619.4 | 750.8 |
| 686 | 769.0 | 867.0 | 1429.7 | 921.6 | 915.8 |

Supplement Figure 1. Acoustic brainstem response (ABR) threshold analyses in normal mice and ototoxicity mice . A) Typical example of ABR measured in normal mouse and ototoxicity mouse in response to click stimuli of indicated intensities. *ABR threshold. B) Quantification results of ABR thresholds did not differ significantly between the normal mouse(n=8) and ototoxicity mouse(n=8) for click. *P<0.0001


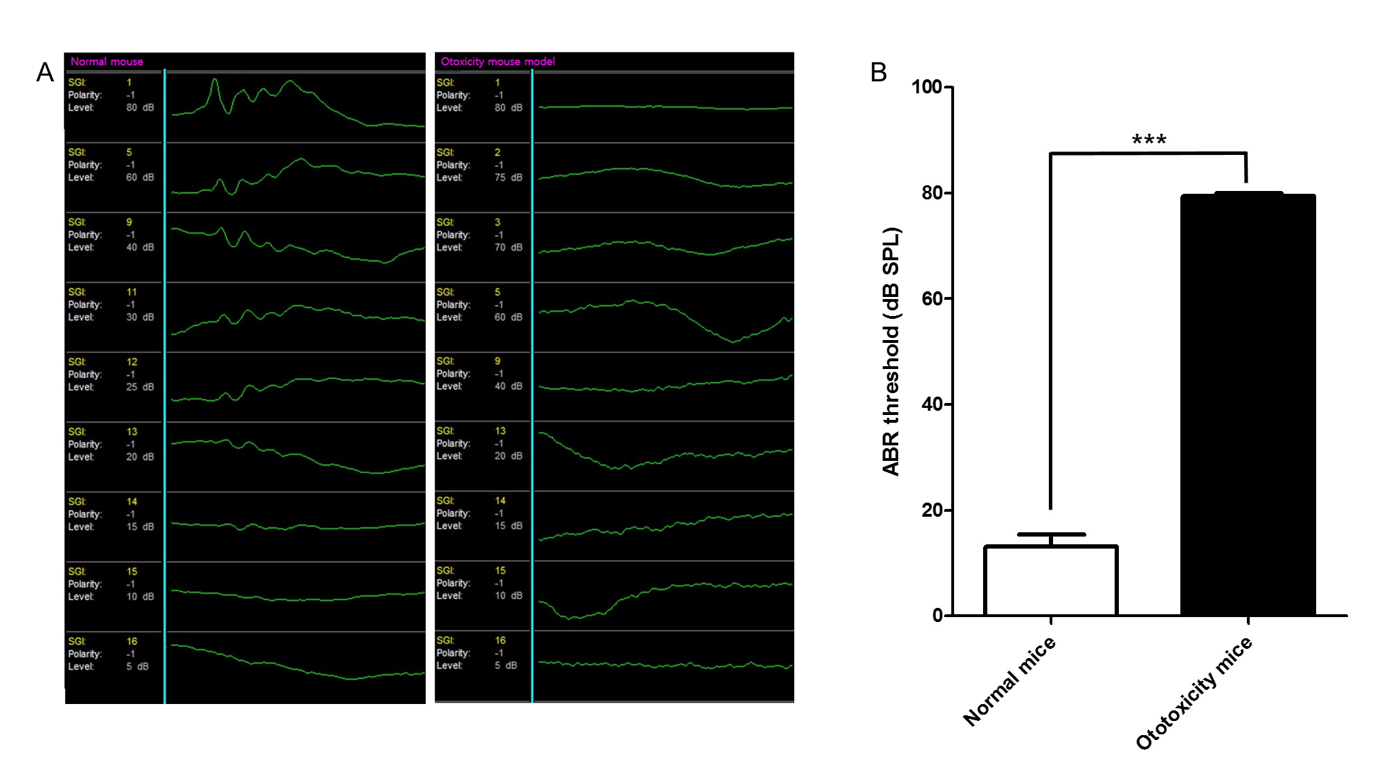

Supplement: Supplementary file 4 [file Table_1.DOC]
